# Nutrient levels within leaves, stems, and roots of the xeric species *Reaumuria soongorica* in relation to geographical, climatic, and soil conditions

**DOI:** 10.1002/ece3.1441

**Published:** 2015-03-06

**Authors:** Mingzhu He, Ke Zhang, Huijuan Tan, Rui Hu, Jieqiong Su, Jin Wang, Lei Huang, Yafeng Zhang, Xinrong Li

**Affiliations:** 1 Shapotou Desert Research and Experiment Station, Cold and Arid Regions Environmental and Engineering Research Institute, Chinese Academy of SciencesLanzhou, 730000, China; 2 Key Laboratory of Stress Physiology and Ecology in Cold and Arid Regions of Gansu ProvinceLanzhou, 730000, China

**Keywords:** Aridity index, carbon, climate, nitrogen, nutrient storage, pH, phosphorus, soil property, stoichiometry

## Abstract

Besides water relations, nutrient allocation, and stoichiometric traits are fundamental feature of shrubs. Knowledge concerning the nutrient stoichiometry of xerophytes is essential to predicting the biogeochemical cycling in desert ecosystems as well as to understanding the homoeostasis and variability of nutrient traits in desert plants. Here, we focused on the temperate desert species *Reaumuria soongorica* and collected samples from plant organs and soil over 28 different locations that covered a wide distributional gradient of this species. Carbon (C), nitrogen (N), and phosphorus (P) concentrations and their stoichiometry were determined and subsequently compared with geographic, climatic, and edaphic factors. The mean leaf C, N, and P concentrations and C/N, C/P, and N/P ratios were 371.6 mg g^−1^, 10.6 mg g^−1^, 0.73 mg g^−1^, and 59.7, 837.9, 15.7, respectively. Stem and root C concentrations were higher than leaf C, while leaf N was higher than stem and root N. Phosphorus concentration and N/P did not differ among plant organs. Significant differences were found between root C/N and leaf C/N as well as between root C/P and leaf C/P. Leaf nutrient traits respond to geographic and climatic factors, while nutrient concentrations of stems and roots are mostly affected by soil P and pH. We show that stoichiometric patterns in different plant organs had different responses to environmental variables. Studies of species-specific nutrient stoichiometry can help clarify plant–environment relationships and nutrient cycling patterns in desert ecosystems.

## Introduction

Within arid ecosystems, high temperatures and low rainfall result in slow biogeochemical cycles of carbon (C), nitrogen (N), and phosphorus (P) (Hartley et al. 2007), low rates of organic matter decomposition, and low primary productivity (Noy-Meir 1973; Belnap et al. 2005). Carbon, N, and P are essential for plant metabolism and growth (Lambers et al. 1998). Under xeric conditions, availability of C and N is related to various processes, such as photosynthesis, N-fixation, and nutrient mineralization (Hartley et al. 2007; Farooq et al. 2012). These processes can be constrained by low water availability and poor soil nutrient conditions (Farooq et al. 2009). Plant C assimilation capacity is reduced under drought stress due to stomatal closure (Medrano et al. 2002), destruction of photosynthetic apparatus, impaired ATP synthesis, and carboxylation enzymes (Farooq et al. 2009). Plant N uptake and fixation in arid ecosystems is reduced by high temperature, low soil moisture (West and Skujins 1978), decreased nitrogen mineralization (Austin et al. 2004), and also affected by fluctuation of soil pH (Hartley et al. 2007). Phosphorus is mainly derived from weathering of primary minerals such as apatite (Belnap 2011). In arid and alkaline soils, availability of P is strongly influenced by CaCO_3_ and high pH (Hartley et al. 2007; Belnap 2011). In response to water and nutrient colimitation conditions, xerophytes may show little plasticity in N:P stoichiometry and keep low tissue nutrient uptake (Drenovsky and Richards 2004). Xerophytes also invest additional C in structural (e.g., cellulose) and defensive (e.g., polyphenols) compounds (Lambers et al. 1998; Sterner and Elser 2002), which result in changes of C:N and C:P ratios in different plant organs (McGroddy et al. 2004).Therefore, understanding the nutrient (C, N, and P) stoichiometry of xerophytes can help predict the biogeochemical cycling in desert ecosystems within the context of global climate change. It can also aid in understanding the homoeostasis and variability of nutrient traits in desert plants.

Nutrient allocation patterns among leaves, stems, and roots reflect the capacity of plants to obtain, transport, and store nutrients (Chapin 1980; Lambers et al. 1998). Thus far, a larger number of studies have been conducted to characterize plant nutrient stoichiometry in a range of geographical areas; however, due to sampling difficulties and labor cost, most these studies were focused on analyzing the nutrient status of leaves, but they did not attempt the same type of analysis on stems and roots (Reich and Oleksyn 2004; Han et al. 2005). In this context, it is important to point out that stems and roots are not simply structural components but also key organs for the uptake, transport, accumulation, and storage of nutrients for plant biosynthesis (Chapin et al. 1990). For example, in woody plants, storage proteins can account for 25–30% of the total extractable proteins and these are stored in structural (cellulose) tissue, bark, and roots of trees (Lambers et al. 1998). These stored nutrients can play an essential role in supporting leaf growth under xeric conditions that are unfavorable for nutrient absorption and CO_2_ assimilation (Lambers et al. 1998). Nutrient resorption is another vital nutrient conservation mechanism in desert plants adapted to water stress and N- and P-limited environments (Drenovsky et al. 2010). It is known that the pattern of N and P conservation differs between plant growth forms (e.g., perennial grass and shrub), but is also mediated by aridity gradients. For example, Bertiller et al. (2005) found that N-resorption efficiency in shrubs decreased with increasing aridity resulting in increase of the N concentration in senescent leaves. Therefore, for the purpose of fully understanding nutrient allocation and storage patterns of desert plants, it is important to study both leaves and storage organs (stems and roots) nutrient conditions.

There are many recent studies on the patterns of foliar C, N, and P stoichiometry and their controlling factors at global (McGroddy et al. 2004; Reich and Oleksyn 2004), regional (Han et al. 2005), and local scales (Zheng and Shangguan 2007; He et al. 2008; Sardans et al. 2011). Patterns of nutrient concentrations in species vary with geography and climate. Different from humid and semihumid regions, foliar N and P levels in arid regions are often negatively correlated with mean annual temperature (MAT) and unrelated to mean annual precipitation (MAP) (He et al. 2014). A substantial amount of plant nutrient data have been reported in global and Chinese studies, but there are few reports from the temperate deserts of China. There are at least two reasons as to why it is necessary to invest more research efforts in to the temperate deserts of China: Firstly, this area contains unique plant resources that are valuable to ecosystem integrity; secondly, it is characterized by a fragile environment subject to the devastating influence of nutrient limitation, overgrazing, and desertification. In addition, it is still unclear how geographical, climatic, and edaphic factors might affect the nutrient stoichiometry for individual desert plant species.

*Reaumuria soongorica* (Tamaricaceae) is a deciduous and xerophytic dwarf shrub, 10–30 (−70) cm tall, much branched; leaves often 4–6 clustered on shortened branches and 1–5 mm × 0.5–1 mm; roots with depths of 0–50 (−100) cm. The shrub is widely distributed across arid regions of China and a dominant species of steppe deserts and typical deserts with limited water and nutrients (Liu 1987). *R. soongorica* plays a vital role in sustaining ecological stability of deserts and providing grazing forage due to its drought-resistant nature (Liu et al. 2007) and abundant foliar levels of proteins, fats, and micronutrients (Hou 1982; Zhou 1990). It represents an opportunity for testing nutrient stoichiometry patterns in one species with a wide distribution. There have been no previous studies on the variation in C, N, and P among different organs of *R. soongorica* in relation to geography (latitude and longitude), climate (MAT, MAP, and aridity index), and edaphic factors (soil organic carbon (SOC), soil total nitrogen (SN), soil total phosphorus (SP), and pH).

Our objectives in this study were to (1) determine the patterns of nutrients (C, N, and P) stoichiometry among leaves, stems, and roots of *R. soongorica*; and (2) evaluate the effects of geographic, climatic, and edaphic factors related to the nutritional status of plant organs within plants growing in different environments.

## Materials and Methods

### Study sites

Samples were collected from 28 desert sites across the Ningxia and Inner Mongolia Provinces in northwestern China (see Table S1 for details). These sites are situated in a region that spans 37.69′ N, 101.56′ E to 40.66′N, 105.72E′, with altitude ranging from 1095 to 1667 m. Mean annual temperature (MAT) ranges are 6.2–9.0°C, mean annual precipitation (MAP) ranges are 107.4 mm to 174.9 mm, and the aridity index (ratio of precipitation to potential evapotranspiration) ranges are 0.09–0.17. Desert soils in this area are saline, alkaline (pH range 7.5–11.2), and limited by low soil organic matter, nitrogen, and phosphorus.

### Sampling and measurement

Plant and soil samples were collected and analyzed during the growing season (August) of 2012. Each sample site was located away from human and grazing animal disturbance. Following a quantitative survey of 10 m × 10 m sampling plot at each site, we selected five individuals of *R. soongorica,* removed them from the soil, and separated the soil from the roots. Plant samples were divided into leaves, stems, and roots, and five replicates of the different organs were combined. We note that the leaves were fully expanded sun exposed and newly matured and the root reached to 50–100 cm soil depth. A total of 84 plant samples and 28 soil samples were collected from the 28 research sites. Leaf, stem, and root samples were rinsed twice with deionized water to remove dust and soil, oven-dried at 60°C for 72 h, and then ground for measurement of C, N, and P concentrations. Carbon and nitrogen concentration in leaves, stems, and roots were measured with a CHNS/O Elemental Analyzer (Perkin-Elmer, USA). Phosphorus in plant organ samples was measured colorimetrically after H_2_SO_4_-H_2_O_2_-HF digestion using the ammonium molybdate/stannous chloride method (Kuo 1996).

Soil samples separated from roots were mixed, air-dried, sieved, and then ground to pass through a 100-mesh sieve. pH was determined by a soil acidity meter (PHSJ-3F, China) using a water extraction method (10 g fresh soil extracted with 50 mL deionized water). For carbon determination, the soil samples were digested in K_2_Cr_2_O_7_-H_2_SO_4_ solution on a heating panel and soil organic carbon (SOC) concentrations were measured by titration (Bao 2000). Soil total nitrogen (SN) was analyzed with a Kjeltec system 2300 Analyzer Unit (Tecator, Höganäs, Sweden). Soil total phosphorus (SP) content was determined with the molybdate/ascorbic acid blue method after digestion with HClO_4_ and H_2_SO_4_ acid (John 1970). The MAT and MAP used in this study were generated using linear interpolation models based on variables of latitude, longitude, and altitude, derived from the climate database from the Inner Mongolia Weather Bureau.

### Data analysis

All data including C, N, P, C/N, C/P, and N/P among leaves, stems, and roots were log-transformed to normalize distributions. We used one-way ANOVA and multiple comparisons to test nutrient differences among leaves, stems, and roots. When organ effects were significant (*P* < 0.05), we used Tukey's HSD post hoc test to compare organ means. We used ANOVAs to test effects of geographical factors (latitude, longitude), climatic factors (MAT, MAP, and Aridity), and edaphic factors (SOC, SN, SP, and pH) on C, N, P, C/N, C/P, and N/P among leaves, stems, and roots. Scatter plots were used to show the relationships among nutrient stoichiometry of plant organs related to geographic factors, and linear or quadratic regression analyses were developed. The relationships among climatic, edaphic factors related to geographic factors were analyzed using linear or quadratic equations. Pearson correlation analyses were used to test correlation among C, N, and P concentrations in different plant organs or among SOC, SN, and ST. All the statistical analysis was conducted using JMP (v.10.0.0; Cary, NC, USA).

## Results

Among the 28 sample sites, the coefficient of variation (CV) for C was less than that for N, P, C/N, C/P, and N/P, indicating that C concentration was less variable than other nutrients (Table1). The mean leaf C, N, P concentrations, and C/N, C/P, N/P ratios were 371.6 mg g^−1^, 10.6 mg g^−1^, 0.73 mg g^−1^, and 59.7, 837.9, 15.7, respectively. Ranges were 313.1–463.2 mg g^−1^, 2.09–21.4 mg g^−1^, 0.16–2.56 mg g^−1^, and 16.9–213.3, 148.1–2685.4, and 6.60–27.0, respectively (Table1). The mean C concentrations in stems (455.8 mg g^−1^) and roots (469.7 mg g^−1^) were significantly greater than that in leaves (*P *<* *0.0001) (Table1). Leaf N concentration was greater than stem N (5.85 mg g^−1^) and root N (5.16 mg g^−1^) (*P *<* *0.0001) (Table1). There was no significant difference in phosphorus concentration (or N/P) among leaves, stems, and roots (*P *>* *0.05). However, both C:N and C:P ratios differed significantly between roots and leaves (*P *=* *0.001) (Table1).

**Table 1:** Organ C, N, and P concentrations and their ratios for *Reaumuria soongorica*. Different letters in the same row indicate significant differences of nutrient concentrations at *P *<* *0.05 using Turkey's post hoc following one-way ANOVA. CV is defined as SD/mean; *n*, denoted number of replicates

Nutrient	Leaf		Stem		Root		*n*	*F* ratio	*P*
	Mean ± SE	CV (%)	Mean ± SE	CV (%)	Mean ± SE	CV (%)			
C (mg g^−1^)	371.6 ± 6.69 **b**	9.53	455.8 ± 3.17 **a**	3.68	469.7 ± 2.55 **a**	2.87	28	138.1	< 0.0001
N (mg g^−1^)	10.6 ± 1.21 **a**	60.5	5.85 ± 0.66 **b**	59.4	5.16 ± 0.56 **b**	57.4	28	11.8	< 0.0001
P (mg g^−1^)	0.73 ± 0.10 **a**	75.5	0.60 ± 0.09 **a**	80.4	0.47 ± 0.08 **a**	86.4	28	2.16	0.12
C/N	59.7 ± 10.1**b**	89.6	128.6 ± 18.4 **ab**	75.5	145.0 ± 20.1 **a**	73.2	28	7.3	0.001
C/P	837.9 ± 118.1**b**	74.6	1339.9 ± 190.0 **ab**	75.0	2005.1 ± 307.2 **a**	81.1	28	7.12	0.001
N/P	15.7 ± 1.01 **a**	33.9	11.2 ± 0.82 **a**	39.0	15.3 ± 2.27 **a**	78.3	28	2.81	0.07

Leaf C was not significantly correlated neither with C in stems and roots, nor with N and P in leaves, stems, and roots (Table S2). Stem C was positively correlated with root C (*r *=* *0.75, *P *<* *0.0001). Leaf N was negatively correlated with root C (*r *=* *−0.43, *P *<* *0.05) and positively correlated with stem N (*r *=* *0.93, *P *<* *0.0001), root N (*r *=* *0.90, *P *<* *0.0001), leaf P (*r *=* *0.68, *P *<* *0.0001), and stem P (*r *=* *0.52, *P *<* *0.01). Positive correlations were found among stem N, root N, leaf P, stem P, and root P (Table S2). Soil organic carbon was positively correlated with SN (*r *=* *0.43, *P *<* *0.05) and SP (*r *=* *0.38, *P *<* *0.05); SN was positively correlated with SP (*r *=* *0.66, *P *<* *0.0001) (Table S3).

**Figure 1:**
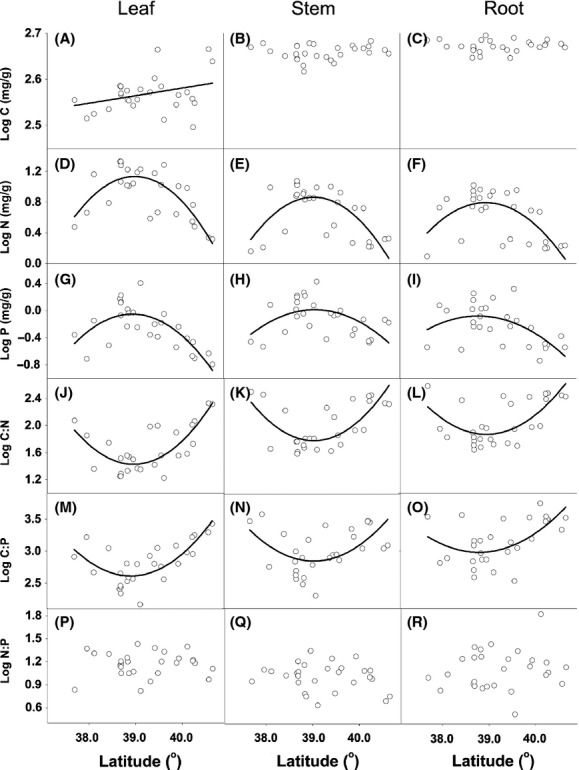
Relationship between organ nutrient stoichiometry and latitude for the *Reaumuria soongorica* across 28 desert sample sites. Data points are log-transformed for the C, N, P, C/N, C/P, and N/P in leaf, stem, and root tissues. Linear fit for (A) leaf C and latitude (*R*^2^ = 0.07, *P *=* *0.09), and quadratic fit for (D) leaf N and latitude (*R*^2^ = 0.58, *P *<* *0.0001), (E) stem N and latitude (*R*^2^ = 0.48, *P *<* *0.0001), (F) root N and latitude (*R*^2^ = 0.42, *P *<* *0.0001), (G) leaf P and latitude (*R*^2^ = 0.53, *P *<* *0.0001), (H) stem P and latitude (*R*^2^ = 0.24, *P *=* *0.01), (I) root P and latitude (*R*^2^ = 0.30, *P *=* *0.005), (J) leaf C/N and latitude (*R*^2^ = 0.57, *P *<* *0.0001), (K) stem C/N and latitude (*R*^2^ = 0.49, *P *<* *0.0001), (L) root C/N and latitude (*R*^2^ = 0.43, *P *<* *0.0001), (M) leaf C/P and latitude (*R*^2^ = 0.56, *P *<* *0.0001), (N) stem C/P and latitude (*R*^2^ = 0.25, *P *=* *0.01), and (O) root C/P and latitude (*R*^2^ = 0.30, *P *=* *0.004) are displayed. Nonsignificant relationships were found for (B) stem C and latitude, (C) root C and latitude, (P) leaf N/P and latitude, (Q) stem N/P and latitude, and (R) root N/P and latitude.

Geographic factors had different effects on the nutrient traits in plants. Latitude had significant effects on the N, P, C/N, and C/P in leaf and root (*P *<* *0.05) (Table2, Fig.1); longitude was not correlated with plant nutrient traits (Table2). Climatic factors had significant effects on the nutrient traits in leaf, but not in stems and roots. MAT, MAP, and aridity had significant effects on leaf N, P, C/N, and C/P (*P *<* *0.05), but no effects on leaf C and leaf N/P (*P *>* *0.05) (Table2). Edaphic factors had different effects on the nutrient traits in plants. Soil organic carbon and SN had no effect on the plant nutrients. Total P level in soil had significant effects on the stem C, N, P, C/N, and C/P and root N, P, C/N, and C/P (*P *<* *0.05), but no effect on stem N/P, root C, and root N/P. The pH level had significant effects on leaf C, stem N, P, C/N, and C/P (*P *<* *0.05) (Table2).

**Table 2:** Summary of ANOVA results (*P*-values) for the effects of environmental factors on the plant C, N, P, C/N, C/P, and N/P in leaves, stems, and roots

Variable		Plant organ																	
		Leaf						Stem						Root					
		C	N	P	C:N	C:P	N:P	C	N	P	C:N	C:P	N:P	C	N	P	C:N	C:P	N:P
Geographical factor	Latitude	*0.09*	**0.02**	**0.01**	**0.02**	**0.01**	0.80	0.53	*0.08*	0.23	*0.08*	0.23	0.40	0.83	**0.03**	**0.02**	**0.03**	**0.02**	0.44
	Longitude	0.84	0.91	0.84	0.89	0.85	0.56	0.24	0.97	0.81	0.98	0.78	0.62	0.64	0.71	0.78	0.73	0.78	0.98
Climatic factor	MAT	0.15	*0.08*	**0.03**	*0.06*	**0.02**	0.61	0.28	0.15	0.22	0.15	0.21	0.84	0.57	0.16	0.24	0.16	0.23	0.98
	MAP	0.15	*0.07*	**0.03**	**0.05**	**0.02**	0.62	0.26	0.14	0.22	0.14	0.22	0.77	0.58	0.14	0.18	0.14	0.17	0.86
	Aridity	0.16	*0.07*	**0.03**	**0.05**	**0.02**	0.62	0.25	0.15	0.23	0.14	0.23	0.79	0.58	0.15	0.19	0.15	0.18	0.87
Edaphic factor	SOC	0.72	0.98	0.98	0.94	0.99	0.91	0.99	0.65	0.92	0.66	0.91	0.39	0.71	0.48	0.73	0.49	0.72	0.80
	SN	0.86	0.32	0.51	0.33	0.51	0.51	1.00	0.23	0.19	0.24	0.20	0.78	0.99	0.18	0.16	0.19	0.16	0.62
	SP	0.95	*0.09*	0.14	0.10	0.15	0.62	**0.03**	**0.02**	**0.03**	**0.02**	**0.03**	0.80	0.61	**0.03**	**0.04**	**0.03**	**0.03**	0.63
	pH	**0.05**	0.27	0.32	0.42	0.45	0.88	0.42	**0.04**	**0.03**	**0.04**	**0.03**	0.64	0.28	0.21	0.88	0.19	0.85	0.24

*P*-values are in bold when *P *<* *0.05 and in italic when *P *<* *0.1.

Leaf C was positively related to latitude (Fig.1). The quadratic regression equations simulated correlations between nutrient traits and latitude (Fig.1). Nitrogen and P concentrations in leaves, stems, and roots increased with latitude up to 39^o^N and then decreased with latitudes above 39^o^N (Fig.1). In contrast, C/N and C/P in leaves, stems and roots were initially decreased with latitudes up to 39^o^N and then increased with latitudes above 39^o^N (Fig.1).

**Figure 2:**
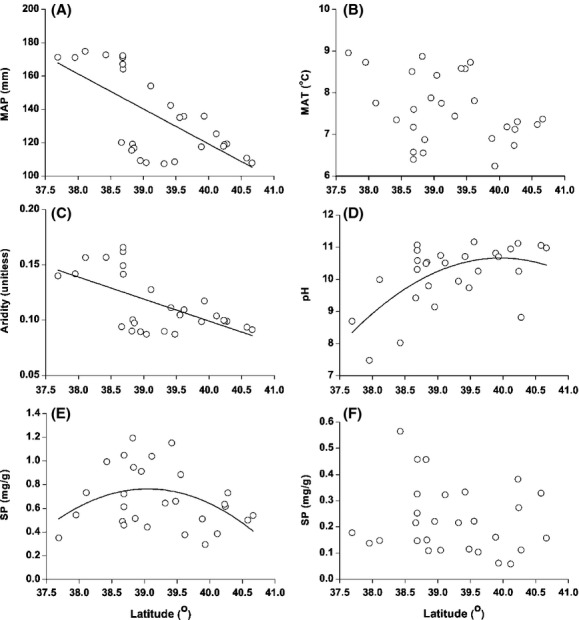
Relationship between climatic and edaphic factors and latitude across 28 desert sample sites. Linear fit for (A) MAP and latitude (*R*^2^ = 0.42, *P *<* *0.0001), (C) Aridity index and latitude (*R*^2^ = 0.34, *P < *0.0001), and quadratic fit for (D) pH and latitude (*R*^2^ = 0.31, *P *<* *0.0001), and (E) SP and latitude (*R*^2^ = 0.17, *P *=* *0.05) are displayed. Nonsignificant relationships were found for (B) MAT and latitude and (F) SP and latitude.

Relationships between climatic, edaphic factors, and latitude are illustrated in Figure2. Mean annual precipitation and the aridity index were negatively and linearly correlated with latitude (*P *<* *0.0001). A quadratic equation was the best fit for the relationship between pH and latitude (*P *<* *0.0001). Soil P increased with increasing latitude up to 39^o^N and then decreased with latitude greater than 39^o^N (Fig.2).

## Discussion

Under arid conditions, drought stress leads to reductions of leaf area, closed stomata, and impaired ATP synthesis and carboxylation enzyme activity. These conditions constrain the photosynthesis activity of desert plants and result in reduced carbon fixation (Barlow 1988; Farooq et al. 2009, 2012). The average N in leaves was 10.6 mg g^−1^, lower than that of the terrestrial flora (20.6 mg g^−1^) (Elser et al. 2000), global flora (20.1 mg g^−1^) (Reich and Oleksyn 2004), and other Chinese shrub species (19.1 mg g^−1^) (Han et al. 2005). Leaf P was 0.73 mg g^−1^ for *R. soongorica*, also lower than that of the terrestrial flora (1.99 mg g^−1^) (Elser et al. 2000), global flora (1.77 mg g^−1^) (Reich and Oleksyn 2004), and other Chinese shrub species (1.11 mg g^−1^) (Han et al. 2005). We suggest that, under arid conditions, *R. soongorica* has limited capacity to assimilate C, N, and P, because of nutrient limitation and reduced metabolic activity. A recent meta-analysis found that drought stress can negatively impact mean concentrations of plant N (i.e., by 3.73%) and P (i.e., by 9.18%) (He and Dijkstra 2014). Reduction of CO_2_ assimilation caused by water limitation has a corresponding effect on plant N and P uptake, due to decreased mass flow, transpiration, and N and P cycles in arid ecosystems (Sardans and Peñuelas 2012). Generally, water limitation in arid regions reduces soil nutrient supply through mineralization (Schimel et al. 2007; Sanaullah et al. 2012), consequently causing decreases in nutrient dissolution and diffusion in the soil (Chapin 1991). Therefore, the extremely low leaf N and P concentrations indicate that both nutrients had limited availability in the habitats occupied by *R. soongorica*.

Leaf C/N and C/P were significantly higher than those of global flora (22.5 and 232) (Elser et al. 2000), grassland flora in China (17.9 and 231) (He et al. 2006, 2008), and the Chinese flora of the Loess Plateau (21.2 and 312) (Zheng and Shangguan 2007). This suggests that arid conditions place greater constraints on N and P content than on C assimilation. Although desert plants tend to have higher N-efficiency, there were limited biological N-fixation (Rundel et al. 1982) and dissolved organic N (Perakis and Hedin 2002), or relatively high N losses as gaseous N (McCalley and Sparks 2009) from desert soils. A recent study demonstrated that gaseous N loss in arid and semi-arid grasslands of China, tends to be higher than the net N accumulation in particular when the aridity index (AI) was below 0.32(Wang et al. 2014). Moreover, plant P concentrations, generally found to be positively related to soil P (Hedin 2004; Han et al. 2005; Chen et al. 2013b), could play a bigger role than plant N in plant growth or ecosystem development (Reich et al. 2009; Vitousek et al. 2010). In our sampling sites, mean soil P was 0.73 mg g^−1^ (range = 0.29–1.19 mg g^−1^) and was greater than the mean value of other Chinese soils (Han et al. 2005). However, most fractions of total soil P in the range of *R. soongorica* were inorganic forms, such as insoluble calcium phosphates, which are less available for desert plant growth (Ma et al. 2009).

The leaf N/P ratio of *R. soongorica* was 15.7, exceeding the 12.7 value of terrestrial flora (Elser et al. 2000) and global flora (13.8) (Reich and Oleksyn 2004), but about the same as in the Chinese flora (16.3) (Han et al. 2005). Due to that the leaf N/P mass ratio is an indicator of N or P constraint in plants (e.g., N and P colimitation when 14 < N/P < 16) (Koerselman and Meuleman 1996), we suggest that growth and distribution of *R. soongorica* in arid regions is largely limited by P. It is to be expected that lower availability of soil P may result from low dissolution of inorganic phosphates (Ma et al. 2009), or from low precipitation, sorption and mineralization (Belnap 2011), or a combination of both. Interestingly, a recent study has demonstrated that in arid climates, drought can decrease plant P concentration (mean decline of 9.18%) more than N concentration (mean decline of 3.73%), which in turn contributes to a higher plant N/P value (i.e., mean increase of 6.98% as compared to nondrought conditions) (He and Dijkstra 2014).

Leaves, stems, and shoots had different nutrient values in *R. soongorica*. Root C and stem C were significantly higher than leaf C (Table1). Under arid conditions, dry matter accumulation is reduced, to varying degrees, in all plant organs. In general, leaves are more easily stressed under xeric conditions compared to stems or roots (Farooq et al. 2012). *R. soongorica* requires adequate water for leaf C assimilation, and it does this through increasing root proliferation, root length, root density, and rooting depth (Shan et al. 2012). In addition, droughts generally have more severe effects on shoots than roots; this increases the dry weight R/S ratio (Farooq et al. 2009). According to our previous studies, the median value of dry weight R/S ratio was 0.97 and ranged from 0.38 to 2.87(Yang et al. 2013). Leaves have higher N but lower C/N and C/P ratios than roots, indicating that different organs allocate available nutrients in different ways. Under N and P limitation, plants allocate proportionally more N and P in photosynthetic structures in an attempt to maintain normal C assimilation and plant growth (Thompson et al. 1997). Besides morpho-anatomical traits (smaller leaves, larger stomata, increased cell wall thickness, etc.), osmotic adjustments through accumulation of organic and inorganic solutes are an effective way to resist drought stress (Serraj and Sinclair 2002). Previous studies focused on the C, N, and P stoichiometry in leaves, but in this study, we found that roots and stems had different nutrient allocation strategies with higher C, C/N, and C/P in roots and higher N in leaves. No differences were found in P and N/P values in different organs.

We found that the relationships of C, N, and P among different organs of *R. soongorica* were quite variable. Leaf C was uncorrelated with N and P concentrations in leaves, stems and roots (with the exception of stem P). Stem C was significantly correlated with root C (Table S2), suggesting that C assimilation differed from N and P fixation. Note that such a result differed from those of several previous studies. For example, McGroddy et al. (2004) found that foliar C was positively correlated with foliar N and P across a sample of worldwide forest flora. In contrast, Zheng and Shangguan (2007) reported a negative relationship between leaf C and leaf N and P among Chinese Loess plateau flora. In our study, we show that C assimilation and allocation in plant organs is decoupled from N and P fixation. Carbon assimilation is limited by CO_2_, water availability, high temperatures, and morpho-physiological traits under xeric conditions while N and P fixation is largely determined by taxonomic traits (Zhang et al. 2012), soil N and P fractions, and availability (Reich and Oleksyn 2004; Farooq et al. 2012; He and Dijkstra 2014). As a resurrection plant from an arid habitat, *R. soongorica* is highly sensitive to unpredictable precipitation and water availability, that is., it generally maximizes C assimilation when water is available. Nitrogen and P supplies are constrained by nutrient mineralization reduction (Schimel et al. 2007), diffusion, and mass flow in xeric soils (Chapin 1991; Lambers et al. 1998). Previous studies have demonstrated that leaf N and P are positively correlated. In the present study, N and P among leaves, stems, and roots were intercorrelated, suggesting that N- and P-related metabolic activities reflect a fundamental feature of basic biochemical processes in plants (Koerselman and Meuleman 1996; Marschner and Marschner 2012).

Many studies have reported responses of leaf nutrient levels to environmental (geographical, climatic, and edaphic) variables at local, regional, and global scales (Reich and Oleksyn 2004; Han et al. 2005; He et al. 2008; Reich et al. 2009). A series of biogeographical and biogeochemical models and hypotheses have be proposed to explain the mechanisms of ecological stoichiometry of plant nutrients in response to global climate change (Hedin 2004; McGroddy et al. 2004; Reich and Oleksyn 2004). Leaf C was not significantly related to latitude (*P *<* *0.09, Fig.1A) but significantly affected by pH (*P *=* *0.05, Table2). Stem C and root C were unaffected by environmental factors (Table2). Soil pH increased with increasing latitude (Fig.2D). In most cases, C allocation can be related to biological and taxonomic characteristics, water, temperature, and nutrient availability (Litton et al. 2007; Chen et al. 2013a), but in arid areas, calcareous soils with a high pH result in low availability of micronutrients (deficiency of Cu, Fe, Zn, etc.) (Welch 1995) and low C assimilation (Lambers et al. 1998). Under high pH conditions, desert plants generally have higher C/N ratios and increased levels of decay-resistant compounds (lignin, cellulose, and tannin) in leaves (Field et al. 1992). Meanwhile, xeric plants reduce leaf transpiration through increasing cuticle and epidermal cell wall thickness which reduces water loss but retains photosynthetic activity (Shields 1950). Leaf N and P were significantly related to latitude with a nonlinear best fit (Fig.1D–F). Leaf C/N and C/P were significantly related to latitude with concave best fit curves (Fig.1G–I). These results are inconsistent with previous studies. Reich and Oleksyn (2004) demonstrated that leaf P and N concentrations (to a lesser degree) of global flora linearly increased with latitude from 43°S to 60°N. Kerkhoff et al. (2005) found that leaf *N* and *P* values across a worldwide sample of plant species were unrelated to latitude. Our analysis indicated that only soil P had a concave-shape relationship with latitude (Fig.2E), so perhaps soil P has an indirect effect on leaf N and leaf P (Table2). Leaf N/P was unrelated to latitude, which is consistent with Chinese flora results presented by Han et al. (2005). One possible reason for these variable results is that the current study occurred within a narrow geographical range. Leaf N/P may be species specific and homeostatic under varying environments (Yu et al. 2010).

Climatic factors had variable effects on plant nutrient values. MAT, MAP, and aridity had significant effects on leaf P, C/N, and C/P (*P *<* *0.05, Table2), and no effects on the leaf C, N/P, and nutrient levels in stems and roots. These results suggest that leaf nutrients are more sensitive to climatic variables than structural organs (stems and roots). With increasing latitude, aridity increased, and MAP decreased (Fig.2A, C). Under xeric conditions, drought stress had significant negative effects on leaf P rather than leaf N (He and Dijkstra 2014), and accordingly, it exerted positive effects on leaf C/N and C/P.

Of the edaphic factors studied, SOC and SN, had no effects on plant nutrient levels. SP had significant effects on stem C, N, P, C/N, and C/P and root N, P, C/N, and C/P (Table2), indicating that SP may be a better indicator of plant nutrient conditions in desert ecosystems than SOC and SN. In xeric areas, SOC is a heterogeneous mixture derived from plant and microbial residues, amino acids, monomeric sugars, cellulose, protein, lignin, etc.(Baldock 2007), and this mixture is influenced by soil water availability, temperature, decomposition, and soil respiration, etc. (Burke et al. 1989; Fierer et al. 2005). Soil nitrogen conditions are based on eolian deposition with nitrate dust and biological assimilation by N-fixing organisms (McNeill and Unkovich 2007). Plant uptake of N is affected by biological properties, soil water conditions, temperature, N-fixation, and N-mineralization. In contrast, soil P is derived from rock weathering and plays an equal or greater role in nutrient uptake limitation of desert plants especially in calcareous soils with high pH (James et al. 2005; Belnap 2011). Our analysis indicates that SP can have significant effects on nutrient conditions of stems and roots but not leaves. Most of the previous studies have focused on nutrient traits in leaves; yet, we found that stems and roots have equal or lower nutrient levels than leaves. Our results suggest that, under conditions of N and P limitation, stems and roots may act as a “nutrient sink” to provide nutrients to *R. soongorica* during growth.

We found quadratic relationships between SP and latitude. SP increased up to 39^o^N but decreased above 39°N (Fig.2E). Aridity was linearly and positively related to latitude (Fig.2C). A study of global drylands demonstrated that increased aridity resulted in greater release of soil P (Delgado-Baquerizo et al. 2013). This could be one reason why SP increased up to 39°N with increasing aridity. However, under greater aridity (>39°N), SP gradually decreased, presumably due to the influence of decreased rainfall, reduced soil water, more precipitation and adsorption, and calcareous soils with higher pH resulting in a higher concentration of calcium phosphate.

In summary, we demonstrated that the plant organs of *R. soongorica* have different nutrient levels. Stems and roots play important roles in nutrient storage and supply in xeric habitats. Nutrient levels in leaves respond to geographic and climatic factors; nutrient levels of stems and roots are most affected by soil P and pH. The study of species-specific nutrient stoichiometry of desert plants is useful for understanding plant–environment relationships and nutrient cycling in desert ecosystems.

## Supporting Information

**Table S1.**Sample sites in the Alxa desert. **Table S2.** Correlation coefficient matrix of C, N and P among leaves, stems, and roots. **Table S3.** Correlation coefficient matrix of SOC, SN, and SP.
