# Supplementary material for: Nutrient levels within leaves, stems, and roots of the xeric species Reaumuria soongorica in relation to geographical, climatic, and soil conditions
Source: Ecol Evol. 2015 Mar 6;5(7):1494–503. doi: 10.1002/ece3.1441 (PMC4395178; doi:10.1002/ece3.1441)
Supplement: Supplementary file 1 — Table S1.Sample sites in the Alxa desert. Table S2. Correlation coefficient matrix of C, N and P among leaves, stems, and roots. Table S3. Correlation coefficient matrix of SOC, SN, and SP. [file ece30005-1494-sd1.docx]

**Appendix**

**Table S1** Sample sites in the Alxa desert

| **Site No.** | **Latitude** | **Longitude** | **Altitude (m)** | **MAT** | **MAP** | **Aridity Index** |
| --- | --- | --- | --- | --- | --- | --- |
| No. 1 | 37°43.23′ | 104°58.42′ | 1338 | 8.96 | 171.29 | 0.14 |
| No. 2 | 37°57.06′ | 105°21.11′ | 1326 | 8.73 | 171.12 | 0.14 |
| No. 3 | 38°25.41′ | 105°43.28′ | 1514 | 7.35 | 172.68 | 0.16 |
| No. 4 | 40°16.64′ | 104°45.69′ | 1313 | 7.31 | 119.43 | 0.10 |
| No. 5 | 40°34.73′ | 104°34.61′ | 1294 | 7.24 | 110.79 | 0.09 |
| No. 6 | 40°39.77′ | 104°33.77′ | 1257 | 7.37 | 107.96 | 0.09 |
| No. 7 | 40°14.46′ | 104°33.25′ | 1366 | 7.12 | 118.88 | 0.10 |
| No. 8 | 40°13.65′ | 104°15.22′ | 1462 | 6.74 | 117.91 | 0.10 |
| No. 9 | 39°53.04′ | 103°38.46′ | 1511 | 6.91 | 117.57 | 0.10 |
| No. 10 | 39°28.76′ | 102°54.91′ | 1275 | 8.57 | 108.59 | 0.09 |
| No. 11 | 39°18.98′ | 101°56.29′ | 1580 | 7.44 | 107.44 | 0.09 |
| No. 12 | 39°02.64′ | 101°57.02′ | 1422 | 6.55 | 119.19 | 0.10 |
| No. 13 | 38°56.68′ | 102°16.35′ | 1344 | 6.88 | 116.72 | 0.10 |
| No. 14 | 38°49.24′ | 102°22.59′ | 1339 | 7.88 | 109.36 | 0.09 |
| No. 15 | 38°39.81′ | 102°16.86′ | 1439 | 8.42 | 108.10 | 0.09 |
| No. 16 | 39°55.73′ | 104°58.32′ | 1564 | 8.87 | 115.55 | 0.09 |
| No. 17 | 39°48.63′ | 105°03.97′ | 1477 | 8.51 | 120.11 | 0.09 |
| No. 18 | 39°33.35′ | 105°24.02′ | 1095 | 7.18 | 125.38 | 0.10 |
| No. 19 | 39°24.98′ | 105°40.72′ | 1129 | 6.24 | 136.03 | 0.12 |
| No. 20 | 39°06.64′ | 105°39.59′ | 1340 | 7.81 | 135.85 | 0.11 |
| No. 21 | 38°41.35′ | 105°38.53′ | 1430 | 8.73 | 135.11 | 0.10 |
| No. 22 | 38°41.19′ | 105°40.50′ | 1514 | 8.58 | 142.41 | 0.11 |
| No. 23 | 38°40.97′ | 105°42.49′ | 1633 | 7.75 | 153.99 | 0.13 |
| No. 24 | 38°40.95′ | 105°43.19′ | 1667 | 7.60 | 164.23 | 0.14 |
| No. 25 | 38°23.81′ | 105°43.46′ | 1539 | 7.17 | 167.16 | 0.15 |
| No. 26 | 38°15.27′ | 105°37.98′ | 1505 | 6.57 | 171.12 | 0.16 |
| No. 27 | 38°06.52′ | 105°30.07′ | 1489 | 6.40 | 172.27 | 0.17 |
| No. 28 | 39°06.74′ | 102°33.48′ | 1536 | 7.76 | 174.90 | 0.16 |

**Table S2** Correlation coefficient matrix of C, N and P among leaves, stems, and roots.

| Nutrient | C (leaf) | C (stem) | C (root) | N (leaf) | N (stem) | N (root) | P (leaf) | P (stem) | P (root) |
| --- | --- | --- | --- | --- | --- | --- | --- | --- | --- |
| C (leaf) | 1.00 | -0.24 | -0.34 | -0.16 | -0.04 | -0.16 | -0.03 | 0.08 | -0.02 |
| C (stem) |  | 1.00 | 0.75*** | -0.29 | -0.33 | -0.32 | -0.29 | -0.41* | -0.02 |
| C (root) |  |  | 1.00 | -0.43* | -0.36 | -0.32 | -0.31 | -0.32 | 0.07 |
| N (leaf) |  |  |  | 1.00 | 0.93*** | 0.90*** | 0.79*** | 0.68*** | 0.52** |
| N (stem) |  |  |  |  | 1.00 | 0.92*** | 0.76*** | 0.72*** | 0.56** |
| N (root) |  |  |  |  |  | 1.00 | 0.72*** | 0.71*** | 0.59** |
| P (leaf) |  |  |  |  |  |  | 1.00 | 0.87*** | 0.63** |
| P (stem) |  |  |  |  |  |  |  | 1.00 | 0.56** |
| P (root) |  |  |  |  |  |  |  |  | 1.00 |

Correlations with *, *P* < 0.05; **, *P* < 0.01; ***, *P* < 0.0001; those without, *P* > 0.05.

**Table S3** Correlation coefficient matrix of SOC, SN, and SP.

| Nutrient | SOC | SN | SP |
| --- | --- | --- | --- |
| SOC | 1.00 | 0.43* | 0.38* |
| SN |  | 1.00 | 0.66*** |
| SP |  |  | 1.00 |

Correlations with *, *P* < 0.05; ***, *P* < 0.0001.
